# Progress in Piezoelectric Nanogenerators Based on PVDF Composite Films

**DOI:** 10.3390/mi12111278

**Published:** 2021-10-20

**Authors:** Yuan Wang, Laipan Zhu, Cuifeng Du

**Affiliations:** 1 School of Civil and Resources Engineering, University of Science and Technology Beijing, Beijing 100083, China; yuanwang@ustb.edu.cn; 2 Beijing Institute of Nanoenergy and Nanosystems, Chinese Academy of Sciences, Beijing 101400, China

**Keywords:** piezoelectric, nanogenerator, PVDF, flexible, energy conversion

## Abstract

In recent years, great progress has been made in the field of energy harvesting to satisfy increasing needs for portable, sustainable, and renewable energy. Among piezoelectric materials, poly(vinylidene fluoride) (PVDF) and its copolymers are the most promising materials for piezoelectric nanogenerators (PENGs) due to their unique electroactivity, high flexibility, good machinability, and long–term stability. So far, PVDF–based PENGs have made remarkable progress. In this paper, the effects of the existence of various nanofillers, including organic–inorganic lead halide perovskites, inorganic lead halide perovskites, perovskite–type oxides, semiconductor piezoelectric materials, two–dimensional layered materials, and ions, in PVDF and its copolymer structure on their piezoelectric response and energy–harvesting properties are reviewed. This review will enable researchers to understand the piezoelectric mechanisms of the PVDF–based composite–film PENGs, so as to effectively convert environmental mechanical stimulus into electrical energy, and finally realize self–powered sensors or high–performance power sources for electronic devices.

## 1. Introduction

The increased consumption of fossil fuels and the ensuing environmental problems call for the development of renewable and clean energy. Over the last few decades, as a kind of active material, piezoelectric materials have attracted more and more attention in applications of self–powered sensors, actuators, and energy–harvesting devices [1,2,3,4,5,6,7]. In 2006, Wang et al. implemented a nanodevice to realize the conversion of mechanical energy into electrical energy by sweeping the metal tip of an atomic force microscope across a vertically grown ZnO nanowire (NW), which was firstly called piezoelectric nanogenerator (PENG) [8,9,10]. Since then, PENGs have become a research hotspot due to their excellent performance of converting tiny and irregular mechanical energy such as vibration, water flow, and body movements into electricity [10,11,12,13,14,15].

**Figure 1:**
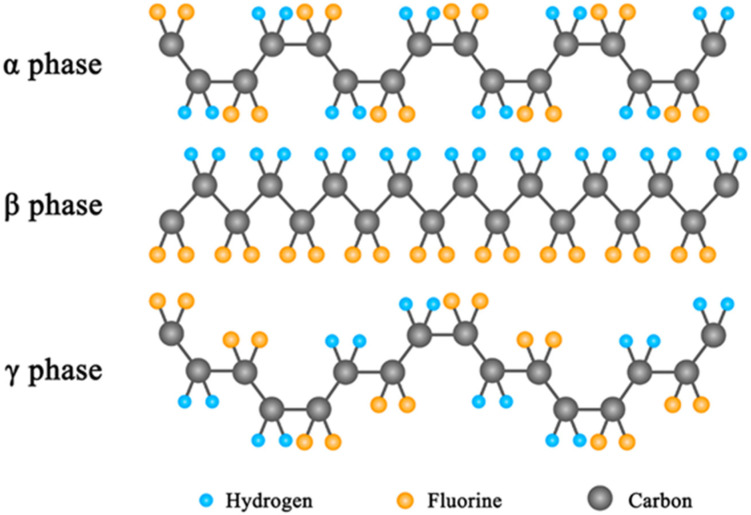
The semi-crystalline α, β, and γ conformations of poly(vinylidene fluoride) (PVDF) [28]. Reproduced with the permission from reference [28].

Normally, although ceramic piezoelectric materials, such as PbTiO_3_ and BaTiO_3_, possess huge piezoelectric coefficients and show excellent mechanical energy–harvesting properties, they are restricted by their rigidity and brittleness [16,17,18,19]. In contrast, piezoelectric polymers possess outstanding characteristics such as good flexibility, light weight, no toxicity, and easy processing [20,21,22,23]. Among them, poly(vinylidene fluoride) (PVDF) is a very promising piezoelectric polymer with long–term stability under a high electric field [24,25,26,27]. PVDF is a kind of semi–crystalline polymer, which mainly crystallizes into five different phases, i.e., α, β, γ, δ, and ε [28,29]. As shown in Figure 1, the α phase is a non–electroactive phase, which results in its nonpolarity, and the γ phase is moderately polarized. The β phase has all–trans (TTTT) conformation, so it behaves as a polar phase, where the C–H and C–F bonds of the PVDF chain in the β phase are distributed at both the ends of the C–C chain separately, leading to an enhanced dipole moment [30]. Therefore, to make PVDF–based PENGs achieve good properties, increasing the content of the β or γ phase is an effective way, which usually uses methods of high–voltage polarization and thermal annealing. However, the piezoelectric coefficient of the PENG is still much lower compared to those of inorganic ceramic materials. Polymer composite piezoelectric materials have fused multiple advantages of the high piezoelectric coefficients of inorganic piezoelectric materials and the flexibility of organic polymers. By adding nanofillers (NFs) into the organic polymer matrix, one can prepare piezoelectric nanocomposites with high piezoelectric properties, good flexibility, and long service life [2,31,32,33,34].

In recent years, nanocomposite films composed of piezoelectric nanoparticles (NPs) and flexible polymers have been intensively studied, which not only improve the piezoelectric properties of polymers but also maintain the flexibility of polymer films. In this paper, the effects of the introduction of various NFs, including organic–inorganic hybrid lead halide perovskites, inorganic lead halide perovskites, perovskite–type oxides, semiconductor piezoelectric materials, two–dimensional (2D) layered materials, and ions, into PVDF and its copolymer structure on their piezoelectric response and energy–harvesting properties are reviewed. The generated electricity is sufficient to drive low–power portable electronic devices and reveal great prospects in self–powered sensors. On the basis of the literature, we retrospect the latest development of flexible PENGs based on PVDF films mixed with different nanocomposites.

## 2. Embedding with Metal Halide Perovskites

### 2.1. Organic–Inorganic Lead Halide Perovskites

Recently, a new organic–inorganic lead halide perovskite material (ABX_3_, A = CH_3_NH_3_ (MA) or CH(NH_2_)_2_ (FA), B = Pb, X = Cl, Br, or I) has attracted wide attention from optoelectronic devices, such as photodetectors, light–emitting diodes (LEDs) and laser diodes, due to their high photoelectric conversion efficiencies, high photoluminescence quantum efficiencies, excellent electrical properties, adjustable emission wavelengths, and high color purity. Researchers have devoted great enthusiasm to the studies of organic–inorganic lead halide perovskite solar cells, and such a kind of cells can realize an energy conversion efficiency of 25.6% (a record high) in a very short time [35,36,37]. Nevertheless, fundamental studies on the piezoelectric properties and applications of organic–inorganic lead halide perovskite materials are still in their infancy. The traditional synthesis process of inorganic piezoelectric materials is complicated and often requires high–temperature calcination and sintering. In contrast, these organic–inorganic lead halide perovskite materials can be prepared by simple low–temperature approaches, such as vapor deposition and solution chemistry [38]. Ding et al. combined FAPbBr_3_ NPs with PVDF polymers to prepare thin films by solution casting and polarize them at an electric field of 50 kV cm^−1^ [39]. The piezoelectric output properties of the composite PENG were significantly improved. Under a pressure of 0.5 MPa, the maximum output voltage and current density of the optimized composite film are about 30 V and 6.2 μA m^−2^, respectively. The proposed PENG can be used to recharge capacitors and light up a red LED through a bridge rectifier.

**Figure 2:**
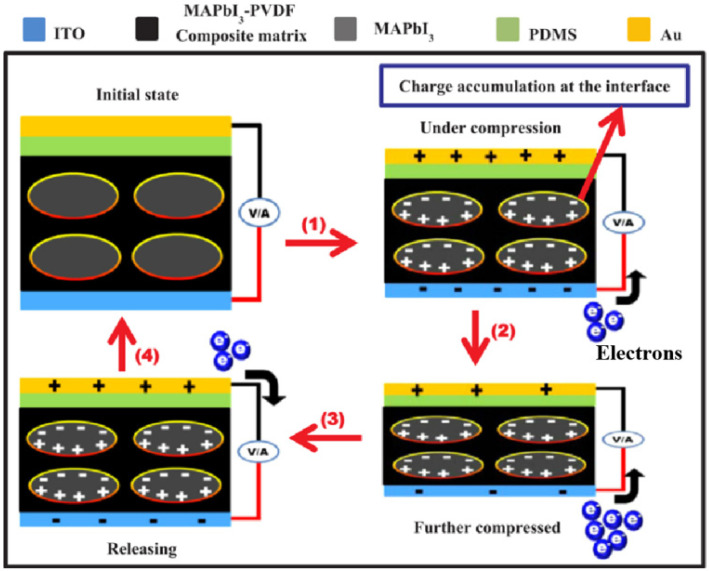
Schematic of the working mechanism of MAPbI_3_–PVDF composite piezoelectric nanogenerators (PENGs) [40]. Reproduced with the permission from reference [40].

**Figure 3:**
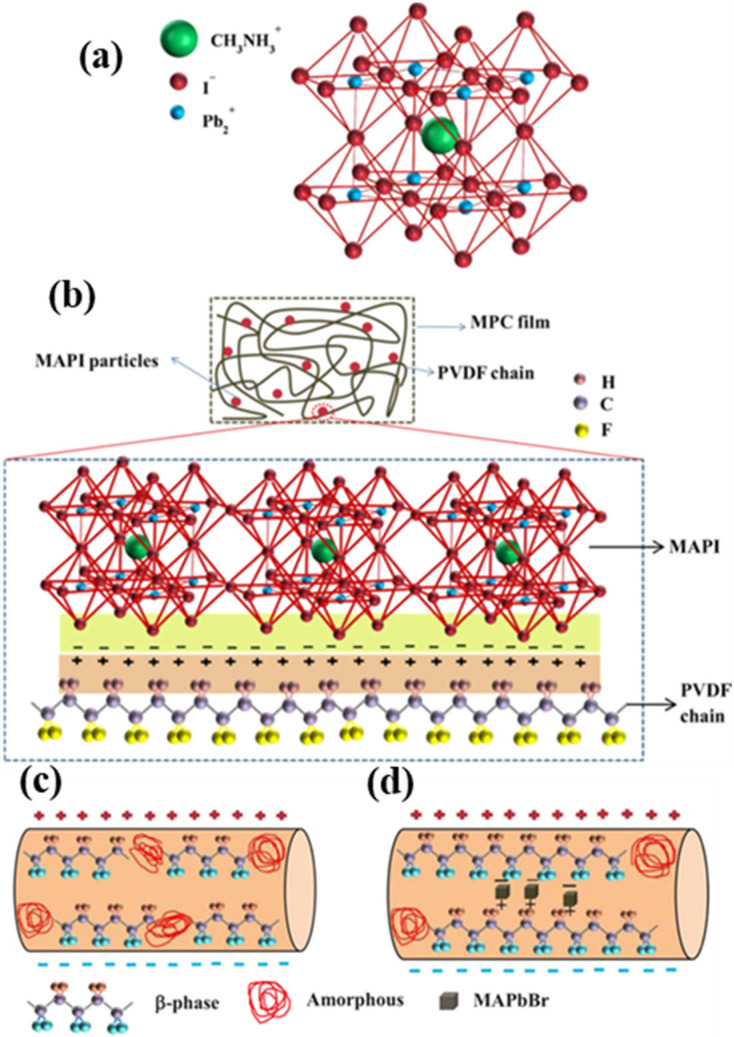
(**a**) The crystal structure of the MAPI perovskite [41]. (**b**) Schematic of the formation mechanism of the electroactive phase in the MAPbI_3_/PVDF composite film [41]. (**c**,**d**) Schematics of the polarization process of the pure PVDF and the MAPbBr_3_/PVDF composite [42]. Reproduced with the permission from references. [41,42].

After that, Jella et al. prepared MAPbI_3_–PVDF composite films by spin coating and tape casting simultaneously [40]. Polarized at an electric field of 80 kV cm^−1^, the open–circuit voltage (V_oc_) and the short-circuit current density (J_sc_) of the PENG prepared by spin coating are 17.8 V and 2.1 µA cm^−2^, respectively, while the V_oc_ and the J_sc_ generated by tape casting are up to 45.6 V and 4.7 µA cm^−2^, respectively. The main reason for the difference in output properties was that the thicknesses of the films prepared by spin coating and tape casting are 6 and 97.7 µm, respectively, which indicates that the film thickness is a key factor affecting PENGs. Meanwhile, the schematic of the power generation mechanism of MAPbI_3_–PVDF composite PENG was illustrated. As shown in Figure 2, no charges are accumulated on the surface of the PENG electrode in the initial stage. When a force is applied to the PENG, the piezoelectric potential produced in the crystalline PVDF causes the filled MAPbI_3_ to form macroscopic dipoles, making charges with the opposite polarity accumulated at the interface and surface of the MAPbI_3_/PVDF composite. Owing to the applied force, the top and bottom electrodes with a reduced charge density allow charges to flow from the bottom electrode to the top electrode, thereby producing a positive signal. In the release process, the relaxation of MAPbI_3_–PVDF matrix aggrandizes the charge density on the two electrodes, making charges flow from the top electrode to the bottom electrode and producing a negative signal. At the same time, Sultana et al. also prepared a MAPbI_3_–PVDF composite film by solution casting and elaborated on the reason for the formation of a stable electroactive phase, i.e., β phase, induced by the addition of a proper amount of MAPbI_3_ to PVDF, as shown in Figure 3 [41]. In the organic–inorganic hybrid lead iodide perovskite compound, the single cationic charge on CH_3_NH_3_^+^ is located on three ammonium hydrogen atoms. This is because in the cage composed of an inorganic PbI_3_^−^ frame, nitrogen atoms have greater electronegativity, as displayed in Figure 3a. The iodine atoms in the PbI_3_^−^ frame are electronegative, which makes the charge density negative, and the negative charge density in turn has an electrostatic interaction with an –CH_2_ dipole and facilitates the nucleation of the electroactive phase, as shown in Figure 3b. A piezoelectric test was conducted on the composite film that was prepared, and a simple finger touch can generate an open–circuit voltage and a short–circuit current of 1.8 V and 37.5 nA, respectively. The devices prepared by MAPbI_3_/PVDF composite films cannot only be used to harvest mechanical energy, but also serve as photodetectors and photosensitive piezoelectric energy harvesters.

PENGs prepared by tape casting or spin coating generally need to be polarized by an external electric field, so as to render piezoelectric properties to the films, while electrospinning can effectively convert mechanical energy into electrical energy without the need of an additional polarization. Sultana et al. synthesized MAPbBr_3_ and introduced it into PVDF nanofibers, which were made by electrospinning process [42]. With repeated finger taps, it is displayed that the open–circuit voltage is 5 V and the short–circuit current is 60 nA. Due to the excellent piezoelectric properties, the nanofibers can also harvest energy from acoustic vibration. Figure 3c,d shows the schematic of the variation mechanism of the β crystalline phase in the electrospinning process of the pure PVDF and the composite film. As shown in Figure 3c, under an applied electric field, PVDF chain can be partially crystallized into the β phase. When MAPbBr_3_ is added into PVDF nanofibers, the charges are induced on the surface of the semiconductor MAPbBr_3_, making electrospinning and in situ polarization generate a greater Coulomb force. This attracts PVDF chains to crystallize on the surface of MAPbBr_3_ in the form of β crystal (Figure 3d) and fulfill the function of a nucleating agent, so that the local amorphous region can be converted into the β crystalline phase. As a result, compared with the pure PVDF, the number of the β crystalline phase in the composite film is increased, and hence, the performances of the PENGs are further improved.

All of the perovskite materials used above are lead–containing materials. They have good properties but are not eco-friendly. Subsequently, Ippili et al. successfully synthesized lead–free MASnI_3_ perovskite under ambient air conditions with a simple anti–solvent–assisted collision technology [43]. The piezoelectric output properties of the polarized MASnI_3_ PENG showed that when a pressure of 0.5 MPa is applied, the open–circuit voltage is about 3.8 V and the short–circuit current density is 0.35 μA cm^−2^. To further enhance the piezoelectric output properties of the MASnI_3_–based PENGs, the authors used an eco–friendly PVDF polymer with a porous structure to synthesize MASnI_3_ films. Polarized at 60 kV cm^−1^, the PENGs based on the PVDF–MASnI_3_ composite material has a maximum output voltage of ~12 V and a current density of ~4.0 μA cm^−2^. Under a pressure/frequency of 0.5 MPa/frequency, the output power of the PVDF–MASnI_3_–based PENGs can drive green LEDs. The output properties of the composite PENGs can remain stable for up to 90 days.

### 2.2. Organic–Inorganic Lead Halide Perovskites

**Figure 4:**
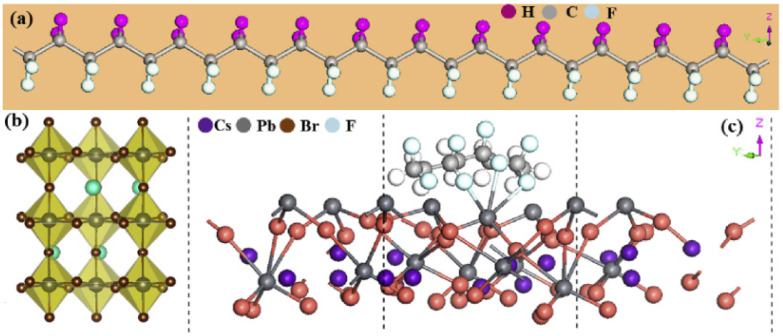
Side views of the PVDF chain (**a**) and the rhombic CsPbBr_3_ (**b**). (**c**) Top view of the CsPbBr_3_–PVDF composite [44]. Reproduced with the permission from reference [44].

Predecessors have also reported extensively on PVDF–enabled PENGs based on all–inorganic perovskite. Mondal et al. illustrated the preparation of all–inorganic perovskite CsPbBr_3_ and PVDF PENGs by using a novel composite material based on a simple solution [44]. The electroactive phase content, crystallinity, piezoelectric coefficient, and optical and energy–harvesting characteristics of the samples were studied. The enhancement of the polar phase of the PVDF matrix was observed by doping CsPbBr_3_. As shown in Figure 4, the piezoelectric responses of all nanogenerators were analyzed by repeatedly hammering in the vertical direction with a pressure of 100 MPa. Among all of the PENGs, the optimal PENG has a maximum output voltage of 120 V and an output current of 35 μA. The excellent properties are attributed to the fact that it contains the most electroactive phases. Figure 4a,b shows the schematic mechanism of the optimized PVDF β–phase chain and the orthorhombic CsPbBr_3_ perovskite structure. As shown in Figure 4c, the PVDF chain bonds to the surface of CsPbBr_3_ via physical absorption. The Pb atoms in the CsPbBr_3_ perovskite are bonded to F atoms in the PVDF chain. In this way, most of the F atoms are located on the same side of the carbon chain, similar to Figure 4a, which results in an improved β phase. Recently, we reported a poly(vinylidene fluoride–trifluoroethylene) (P(VDF–TrFE)) PENG doped with inorgarnic CsPbBr_3_ quantum dots, and a remarkable improvement in piezoelectric output performance was revealed due to the increased β crystalline phase of the P(VDF–TrFE) [45]. Based on a one–step electrospinning method of solutions including CsPbBr_3_ precursors and PVDF, we also fabricated CsPbBr_3_@PVDF composite nanofibers using an in situ growth technology, and the composite PENG displays better thermal/water/acid–base stabilities [46]. The properties of the PVDF halide–based perovskite PENGs are shown in Table 1 below, where RL represents corresponding resistive load when measuring the power output.

## 3. Embedding with Perovskite–Type Oxides

### 3.1. Lead Zirconia Titanate (PZT)

**Figure 5:**
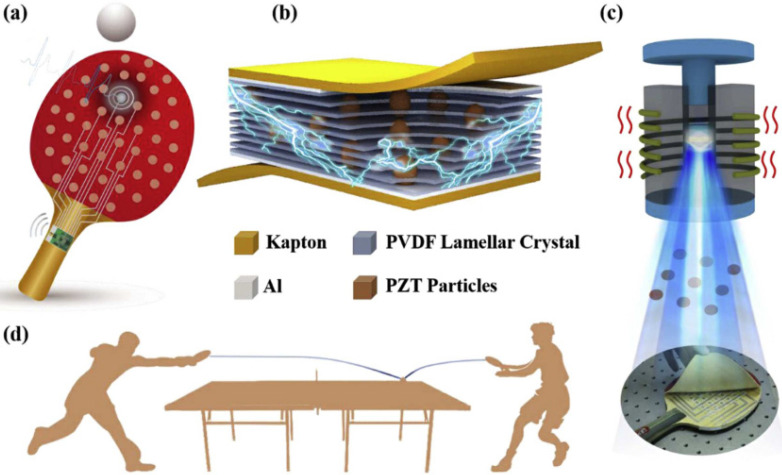
Schematic of the intelligent training system [48]. (**a**) Schematic of the intelligent rackets for table tennis monitoring. (**b**) The overall diagram and the detailed structure of the sensor. (**c**) The piezoelectric composite material of the intelligent racket and the production process of optical photos. (**d**) The application of the intelligent table tennis racket. Reproduced with the permission from reference [48].

Wankhade et al. made an energy–harvesting device that is easy to manufacture, durable, cheap and efficient, by using a PVDF and PZT nanohybrid system [47]. A self–powered piezoelectric sensor (PES) based on a PZT/PVDF composite was reported for a high–quality individual training guidance on table tennis [48]. As shown in Figure 5a, the intelligent table tennis racket is composed of three parts: a PZT/PVDF composite material, a signal processor, and a wireless transmission module. Among them, the sensor array made of the PZT/PVDF composite material is evenly distributed on the racket, and the signal processor and the wireless transmission module are fastened on the handle of the racket (Figure 5b). In this device, the layered structure can help release stress and respond quickly to external forces. In addition, the potential accumulation of different layered crystals makes them more sensitive to pressure. Thus, in order to obtain a piezoelectric material with this structure, they adopted a simple two–step nonsolvent-induced phase separation combined with a hot press process (Figure 5c). A sensor array is integrated on the racket to detect table tennis signals, as shown in Figure 5d, and quantitatively obtain the hitting position and the contact force. More importantly, the sensor has a series of excellent characteristics, such as a sensitivity of up to 6.38 mV N^−1^, a response time shorter than 21 ms, and a good stability.

PZT–based PENGs have a high power generation capacity owing to their high piezoelectric coefficient, but they are faced with global limitations due to the harm of lead toxicity to human health, biocompatibility, and environmental pollution. For this concern, the embedding of eco–friendly and biocompatible PENG materials based on lead–free piezoelectric ceramics such as barium titanate (shorted as BT or BTO) or potassium sodium niobate (KNN) has attracted more and more attention for replacing PZT.

### 3.2. Barium Titanate (BaTiO_3_, also abbreviated as BT or BTO)

**Figure 6:**
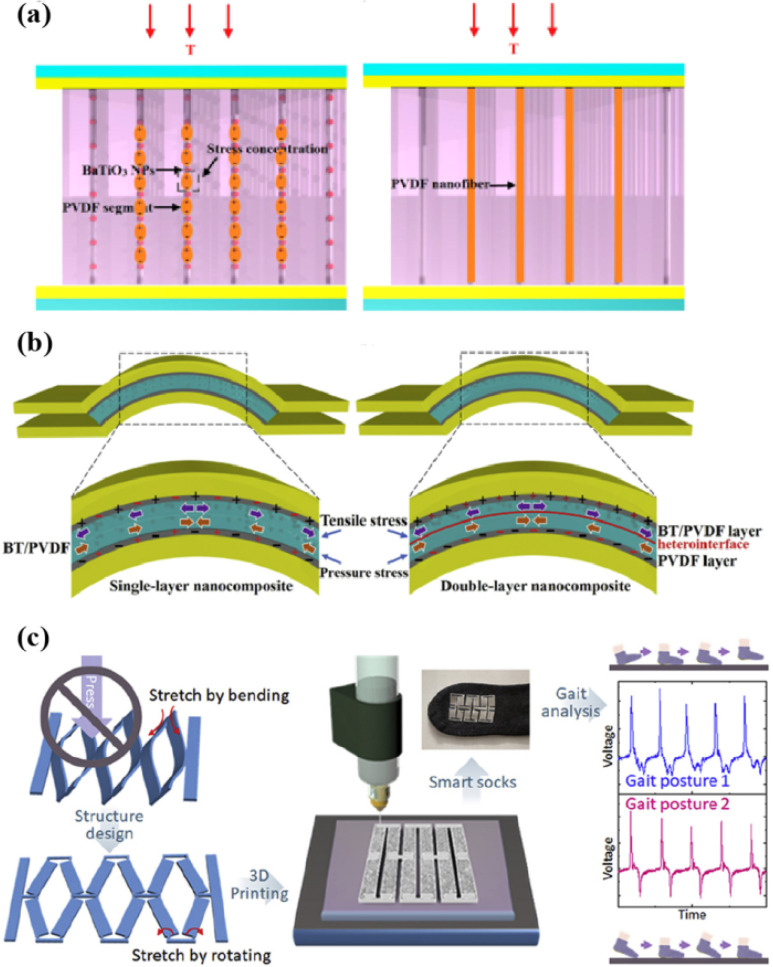
(**a**) Schematic of a vertical stress applied to composite nanogenerator (NG) and pure PVDF PENG [49]. (**b**) Schematic of distributions of stress and related induced charges in the single–layer and double–layer BaTiO_3_ (BT)/PVDF nanocomposite films [50]. (**c**) Schematics, image, and gait analysis of the fully printed PENG installed on socks [51]. Reproduced with the permission from references [49,50,51].

In 2014, Zhao et al. reported a high–performance flexible composite PENG based on BaTiO_3_ NPs and a PVDF composite film (BaTiO_3_ NPs@PVDF) by a solvent evaporation method [49]. The BaTiO_3_ NPs are evenly dispersed in the fibrous PVDF matrix and form a directional structure without aggregation. Compared with the pure PVDF, the BaTiO_3_ NPs not only raise the piezoelectric potential of the PVDF, but also increase the local stress of PVDF, giving a greater deformation to the polymer. Figure 6a shows the schematic of the composite PENG and the pure PVDF PENG under a vertical stress. For the composite film, PVDF fibers are divided into many segments by the BaTiO_3_ NPs. When the film is subjected to a compressive stress, NPs can be used as a concentration point of stress. For this reason, the local deformation of PVDF segments increases significantly and generates a great piezoelectric potential, while the deformation of the pure PVDF PENG does not increase. Therefore, the piezoelectric output generated by the composite PENG would be greater than that of the pure PVDF PENG. Under a pressure of 10 MPa, the maximum open–circuit voltage and the short–circuit current obtained are 150 V and 1500 nA, respectively.

Alluri et al. replaced the Ti^4+^ position of BT nanocubes with different doping ratios of Zr^4+^, to promote the properties of the PENG [52]. By using the molten–salt growth method and replacing the Ti^4+^ (0.605 Å) site with a Zr^4+^ atom (0.72 Å), they modified high–purity BaTiO_3_ nanocubes (without changing the quadrilateral structure). The piezoelectric coefficient can grow from 100 pC N^−1^ (BaTiO_3_) to 174–236 pC N^−1^ (BaTi_(1 − x)_Zr_x_O_3_), and it also holds a better electromechanical conversion efficiency for energy harvesting. Moreover, the use of the BaTi_0_._9_Zr_0_._1_O_3_/PVDF–based PENG as an active sensor was also demonstrated. The electrical output of this PENG can estimate its input flow velocity (inertial force) through the outlet pipeline. Shin et al. reported a flexible composite film PENG composed of hemispherically polymerized BaTiO_3_ nanosheets (NSs) and poly(vinylidene fluoride–hexafluoropropylene) (P(VDF–HFP)) [53]. A solvent evaporation method introduced here is simple, economical and suitable for large–scale manufacturing of high–performing flexible PENGs. Afterwards, Chen et al. put forward a PENG based on piezoelectric P(VDF–TrFE)/BaTiO_3_–reinforced nanocomposite microcolumn arrays, which are made by a reliable and scalable nanoimprint lithography process [34]. At a press of 50 N, the output was 7.3 times larger than that of the original P(VDF–TrFE) film. The weak interface and the poor dispersion of fillers seriously restrict further enhancement of their electromechanical properties. Therefore, Shi et al. adopted a hydrothermal method to fabricate BaTiO_3_ NWs as piezoelectric reinforcing fillers, grafted a layer of high–modulus poly(methyl methacrylate) (PMMA) onto the surface of the NWs by surface–initiated polymerization and used electrospinning to prepare PMMA@BaTiO_3_/P(VDF–TrFE) fiber PENGs [17]. The PMMA is grafted onto the BaTiO_3_ matrix, so that the NWs can be evenly dispersed into the P(VDF–TrFE) matrix, thereby strengthening the stress transfer at the interface between the piezoelectric NWs and the P(VDF–TrFE) matrix and significantly promoting the output properties of the PENGs. Later on, someone studied the strategy of realizing a synergistic effect by preparing different composite layers and fabricated a double–layer (DL) PVDF–based nanocomposite composed of a semi–high–content BTO NP layer, which performs well over those made with single–layered (SL) films [50]. The stress distribution is shown in Figure 6b. In a homogeneous SL film, the opposite stress states applied to the two halves of the film can generate opposite induced charges and lower the overall apparent piezoelectric response. In a nonuniform DL film, due to the different polarization modes and dipole orientations of the BTO/PVDF layer and the neat PVDF layer, the offset effect is weak, and a great net pressure response can be realized. The charges accumulated on the interlayer between the BTO/PVDF layer and the PVDF layer contribute greatly to the improvement of the output. The DL film containing 20 vol% BTO NPs has an excellent output voltage of 6.7 V, an output current of 2.4 μA, and a stability within 3% over 1000 circles.

With the development of wearable electronic devices, stretchable energy harvesters hold great appeal due to their ability to minimize the need to recharge wearables frequently. Zhou et al. investigated the preparation of a telescopic kirigami PENG based on BaTiO_3_ NPs, a P(VDF–TrFE) matrix, and a silver film–based electrode, by using an all three–dimensional (3D) printing process [51]. Such a kind of PENGs can be stretched to a strain of more than 300%, and the device can be installed on socks and other wearable textiles in order to form energy harvesters, collect treading energy from feet and serve as a self–powered gait sensor (Figure 6c). Subsequently, many researchers employed modified BTO, such as graphene NSs, polydopamine, and silver NPs, to improve the properties of PVDF–based PENGs [16,54,55].

### 3.3. Potassium Sodium Niobate (KNN)

PZT has a high piezoelectric coefficient (d_33_: ~200–750 pC N^−1^) and a high Curie temperature (T_C_: ~180–230 °C). Nonetheless, the lead content of PZT is more than 60%, and it is not eco–friendly. To solve this problem, in recent years, people have been studying KNN. KNN has a higher dielectric constant, a higher coupling coefficient, a higher Curie temperature, and a higher piezoelectric coefficient (d_33_: ~700 pC N^−1^) [56]. What is more, it is a completely lead–free material, so it does not have environmental problems as PZT. However, it possesses a very high rigidity and a low breakdown strength, which limits its applications in many piezoelectric fields. Many researchers combined it with the piezoelectric material PVDF and its copolymer. In doing so, not only the piezoelectric properties can be promoted, but also the flexibility can be enhanced. Teka et al. tried using a nanocomposite flexible fiber mesh based on the nanostructure of the PVDF/KNN composite to manufacture a simple and unique lightweight energy harvester, which is polarized in situ by an electrospinning technology [57]. There are also some similar reports. For example, a nanogenerator composed of lead–free KNN nanorods (with a length–diameter ratio of 8.5) and PVDF outputs an open–circuit voltage of 17.5 V and a short–circuit current of 0.522 μA [58]. After that, someone investigated a fiber–mesh PENG, which is electrospun by silane–modified KNN doped with PVDF [59]. It was found that the surface–modified KNN (SM–KNN) of the PVDF–doped nanofiber mesh can improve piezoelectric properties, because it can cause the depolymerization of NFs and further induce better interactions between KNN nanorods and PVDF polymer chains. Vivekananthan et al. developed a kind of lead–free piezoelectric NPs (1− x)K_0.5_Na_0.5_NbO_3–x_BaTiO_3_ by substituting BTO NPs into the KNN lattice [60]. When x ≈ 0.02, the piezoelectric properties of the KNN lattice can be enhanced without changing the orthorhombic phase of the KNN lattice, and the lattice is impregnated in the PVDF matrix. At a low mechanical force of ~0.4 N, the maximum open–circuit voltage generated by the composite film nanogenerator (x ≈ 0.02) is 160 V, and the instantaneous area power density is ~14 mW m^−2^. A performance comparison of the PVDF–based metallic oxide perovskite PENGs is shown in Table 2.

## 4. Semiconductor Piezoelectric Materials

Bhavanasi et al. reported the enhanced piezoelectric energy–harvesting properties of the DL film of polarized P(VDF–TrFE) and graphene oxide (GO) [61]. The increased voltage and power output in the presence of GO films are ascribed to the electrostatic contribution of GO, residual tensile stress, the improved Young’s modulus of the DL film, and the existence of the interfacial space charges between the P(VDF–TrFE) and the GO film. Some researchers reported a kind of flexible, self–powered and multifunctional electronic skin, which can power the whole system by integrating three types of sensors to monitor environmental signals and four micro supercapacitors [62]. All of the sensors and micro supercapacitors are made of the reduced GO (rGO)–on–PVDF nanofibers. The pressure sensing part can monitor wearers’ biological signals, such as their neck pulses, saliva swallowing, voices, and body movements.

Choi et al. reported an analysis of the power enhancement of hybrid piezoelectric structures composed of zinc oxide (ZnO) NWs and PVDF polymers, conducted a test on its mechanical properties by an atomic force microscope and simulated it by the finite element method [63]. The electrical properties of the hybrid nanogenerator were observed with an electrostatic force microscope and through direct I–V measurements. Based on this analysis, the internal strain of ZnO NWs was transferred to PVDF in the hybrid structure, thereby enhancing the power output of the hybrid nanogenerator. These results provided a new approach to optimizing the design of the hybrid piezoelectric structure and the spatial arrangement of nanostructures. Many researchers have realized applications related to ZnO/PVDF composite films. By taking the ZnO nanoneedle/PVDF composite film as the material, Shin et al. successfully prepared a highly sensitive, wearable and wireless pressure sensor. The addition of ZnO nanoneedles as the nucleating agent improves the crystallinity, dielectric constant, and elastic modulus of PVDF–based composite films. Moreover, highly conductive rGO is used as electrodes for pressure sensors and Bluetooth antennas via screen printing [64]. The wireless signals of heart rates are monitored sensitively without any distortion and delay. Compared with pressure sensors made of wires, they have a similar oscillation. These characteristics suggested that such a kind of ZnO nanoneedle/PVDF film is a suitable candidate for commercial and continuous wireless pressure sensors and can be used for the purpose of health monitoring and intensive care. Other researchers synthesized flexible ZnO NPs–impregnated PVDF films with excellent piezoelectric, dielectric, and optical properties in situ [65]. Two simple prototype energy–harvesting units based on ZnO–PVDF thin films are fabricated. One is a PENG and touch sensor, which was named ZPENG, and the other is a self–charging pro power battery (PPB) pack. ZPENG can collect electricity from our living system and nature by virtue of superior properties. What is more, the self–charging PPB made also shows a higher efficiency than other hybrid optical supercapacitors (e.g., other photovoltaic cells with storage functions that have been reported so far). Later on, someone put forward a flexible self–powered ZnO/PVDF/fabric electronic skin, which has many functions such as tactility, atmosphere detection, and self–cleaning [66]. The piezoelectric PVDF and tetrapod ZnO (T–ZnO) nanostructures are hybridized on the flexible fabric substrate. The piezoelectric, air–sensitive, and photocatalytic properties of T–ZnO nanostructures are banded together. The piezoelectric effect of the T–ZnO/PVDF composite gives rise to motion–driven tactile behaviors. The structure of this new material system can facilitate the development of the flexible self–powered multifunctional electronic skin. The interactive human–machine interface (iHMI) is a bridge between men and robots, which has important demands for changes in perceived pressure and bending angle [67]. Deng et al. designed a flexible self–powered PES based on PVDF/ZnO nanofibers and used it for remote gesture control in iHMI [68]. The designed PES could work in both bending and compacting modes, and the sensitivity of the PES can be adjusted with the ratio of ZnO to PVDF. Within the range of 44°–122°, an optimal bending sensitivity of 4.4 mV deg^−1^ and a fast response time of 76 m can be obtained. On this basis, the as–prepared flexible PES is installed on a spring collet and a book, to detect the clamping force and the bending angle in the opening and closing stages.

**Figure 7:**
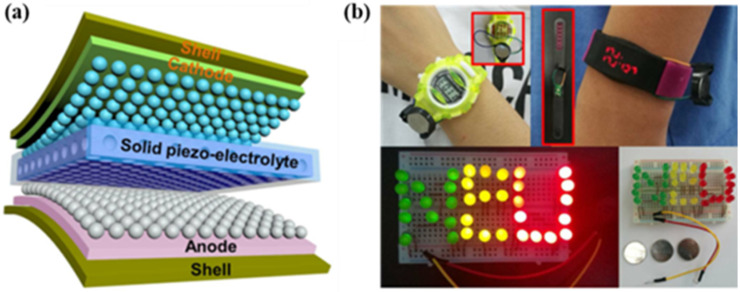
(**a**) The design principle of the all–solid–state self–charging power cell (SCPC). (**b**) Application of all–solid–state SCPCs, such as powering smart watches, sports bracelets, and LEDs. Reproduced with the permission from reference [69].

By taking a mesoporous PVDF–LiPF_6_ film as the piezoelectric electrolyte, He et al. prepared a new all–solid–state self–charging power cell (SCPC) [69]. The solid piezoelectric electrolyte can serve as both an electrolyte and a piezoelectric diaphragm. As shown in Figure 7a, the all–solid–state flexible SCPC can be effectively charged by means of mechanical deformation, to directly obtain/store the kinetic energy of the body. The SCPC sealed in a stainless steel battery can be charged by compressive deformation (30 N, 1 Hz), and the storage capacity within 240 s is 0.118 μA h, which is about 5 times that of the traditional nonintegrated system. The SCPC sealed in a flexible shell can be charged by bending deformation. Such a kind of all–solid–state flexible SCPCs can power most wearable electronic devices, including sports bracelets, smart watches, and LED lamps, as shown in Figure 7b. This study provides an innovative way to develop self–sustainable batteries and self–powered wearable electronic devices.

Studies on PENGs based on boron nitride nanotubes (BNNTs) are still blank. Ye et al. reported the development of a PENG with excellent output properties and outstanding neutron radiation shielding ability by using microstructured BNNTs–doped P(VDF–TrFE) nanocomposites [31]. This PENG achieves a good flexibility, a high performance, and an excellent radiation resistance under harsh conditions. Compared with films without BNNTs, the doped PENG has a neutron radiation shielding effect of 9%, and the neutron cross–section grows by 260%. After 2 h of neutron irradiation, it still maintains a high output. Meanwhile, at a pressure of 0.4 MPa, the output voltage of the nanocomposite PENG is 22 V, and the sensitivity is 55 V/MPa, which is 11 times that of the original P(VDF–TrFE) film. This remarkable property enhancement can be ascribed to the synergic contribution of strong piezoelectric BNNTs and the strain constraint effect of nanocomposite microstructures. Simultaneously, PENGs based on P(VDF–TrFE) doped with GeSe NSs were also studied [70]. When the mass fraction of GeSe NSs is only 4 wt % at a pressure of 50 N cm^−2^, the maximum open–circuit voltage density is 17.58 V cm^−2^, the short–circuit current density is 1.14 µA cm^−2^, and the power density is 9.76 µW cm^−2^. Furthermore, a new multifunctional nanocomposite film was developed by integrating piezoelectric molybdenum disulfide (MoS_2_) and PVDF polymers [71]. Wasted mechanical energy can be used to effectively generate power and purify water. The embedded MoS_2_ NPs have long–term activity, recyclability, and ability to treat a large amount of water, without the spillage of NPs. Under dark conditions, the prepared film exhibits an efficient, rapid and stable pressure–sensitive degradation effect on all of the four toxic and carcinogenic dyes (>90% within 20 min). It can be reused (at least 10 times) without any loss of catalytic activity. Recently, we also demonstrated that a micropillar structure and solvent treatment can greatly enhance the output of NSs–doped PENGs [72,73]. A performance comparison of the PVDF–based PENGs doped with semiconductor piezoelectric materials is summarized in Table 3.

## 5. Rare Earth Ions

**Figure 8:**
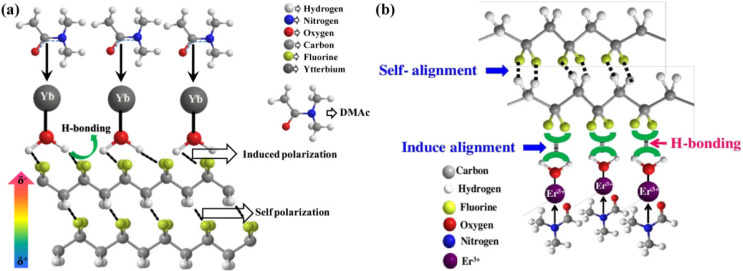
(**a**) The self–polarization process induced by the hydrogen bond interaction between Yb^3+^–PVDF film molecules and the dipolar interaction between Yb^3+^ salt and the PVDF chain [74]. (**b**) Schematic of the β phase nucleation process in the Er^3+^/PVDF film [75]. Reproduced with the permission from references [74,75].

This section may be divided by subheadings. It should provide the concise and precise descriptions of experimental results, their interpretation, as well as experimental conclusions that can be drawn. Ghosh et al. added rare earth ytterbium (Yb) salt with hygroscopicity to PVDF, implemented a porous composite film with a locally oriented –CH_2_/–CF_2_ dipole and avoided the traditional polarization process [74]. The piezoelectric output of the PENG is achieved by the synergy of the self–polarized –CH_2_/–CF_2_ dipole with an electret–like porous structure in the composite films. In the PVDF–DMAc solution doped with Yb^3+^ salt, DMAc solvates PVDF and forms a resonant structure with Yb^3+^. Meanwhile, due to its polar aprotic and hydrophilic properties, it gradually absorbs moisture in the atmosphere. Therefore, water molecules encircle the F ion of PVDF. In addition, there is a strong hydrogen bond interaction between the water molecules and the F iron in PVDF, which makes the –CH_2_/–CF_2_ dipole in PVDF unidirectional, as shown in Figure 8a. As a result, the output as a mechanical energy harvester is greatly enhanced.

Similar to the above experiment, a new infrared–sensitive Er^3+^–modified PVDF film can convert mechanical energy and thermal energy into useful electrical energy [72]. The addition of hygroscopic erbium salt Er^3+^ to PVDF can form a self–polarized piezoelectric β phase, as well as an electret–like porous structure, so as to improve the piezoelectric properties. As shown in Figure 8b, after long–term stirring, Er^3+^ salt doped in a PVDF–DMF solution gradually absorbs moisture in the atmosphere, because of its polar aprotic and hydrophilic properties. Accordingly, the water molecules from moisture and the coordinated water in Er^3+^ salt (ErCl_3_, 6H_2_O) interact and encircle the F^−^ ion in PVDF. Therefore, the F^−^ strong hydrogen bond interaction (O–H––F–C) between water molecules and F^−^ in PVDF powers the –CH_2_/–CF_2_ dipoles and arranges them in an all–trans (TTTT) conformation (i.e., β phase). What is more, it was also proved that due to its excellent infrared absorption properties, Er^3+^ enhances the heat transfer to the Er^3+^/PVDF film, causes rapid temperature fluctuations and improves the conversion of heat release energy. The superior mechanical sensitivity (~3.4 V Pa^−1^) of the PENG enables it to design a wearable self–powered health monitoring system through man–machine integration.

## 6. Other NFs

**Figure 9:**
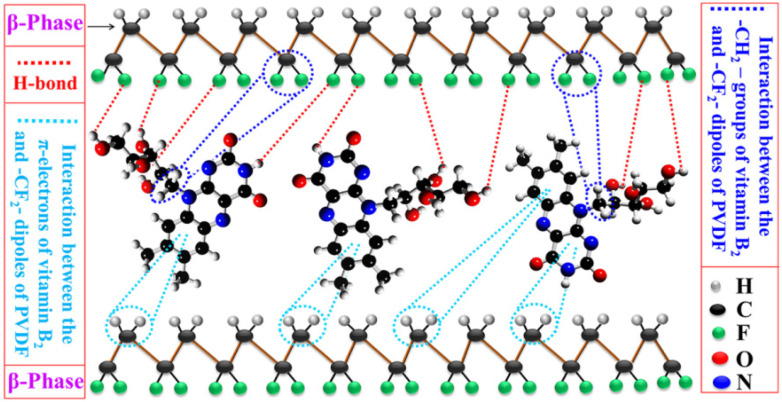
Schematic of various interactions between the PVDF dipole and vitamin B_2_ (VB_2_) [76]. Reproduced with the permission from reference [76].

Karan et al. reported a PVDF–based multifunctional advanced energy material assisted by a bioinspired vitamin B_2_ (VB_2_). By fusing VB_2_ and PVDF, they designed a PENG entirely based on organic and biological compatibility [76]. This PENG can collect multiple energies (mechanical/acoustic/wind) simultaneously. As an effective β phase (~93%) PVDF stabilizer, VB_2_ is introduced into the field of energy harvesting. The existence of the polar β phase can be related to the interaction between –CH_2_^−^/–CF_2_^−^ dipoles in PVDF and delocalized π electrons in carbonyl and amino groups in VB_2_. It is also possible that the F/H atoms of PVDF are attracted by the functional groups (–OH, carbonyl, etc.) in VB_2_ through the H bond/electrostatic force interaction (Figure 9). Through the surface charge–induced polarization, the polar surface with opposite charges on VB_2_ interacts with different PVDF dipoles (–CF_2_^−^/–CH_2_^−^). Consequently, negative and positive charge densities can be obtained on the surface of the composite material, which is helpful build a piezoelectric–sensitive polarized β phase.

A metal foil electrode or a metal electrode coated with film is taken as the charge harvester. Due to huge mismatches of Young’s modulus and Poisson’s ratio between the electrode and the active piezoelectric material, the service life of the device under extended cyclic stress is diminished. For this reason, Ghosh et al. designed an all–fiber wearable high–performance PENG, in which platinum (Pt) NPs and one–dimensional PVDF NF arrays (Pt–PVDF NFs) with highly aligned interfaces are sandwiched between flexible conductive fabrics composed of arrays of interlocking microfibers [77].

## 7. Self–Poled PVDF-Based PENGs with Long–Term Stability

PVDF–based PENGs with embedded NFs own enhanced energy harvesting properties; however, they usually require a poling process to promote the piezoelectric properties and suffer from the long–term stability of the electrical outputs [78]. Therefore, self–poled PVDF–based PENGs with long–term stability are much desired. Recently, Thakur et al. made a self–poled PENG based on a ZnO/PVDF thin film with a long–term stability of at least 8 weeks [65]. Pusty et al. reported a flexible self–poled PENG based on a reduced graphene oxide–silver (rGO–Ag) NP /PVDF film [79]. The PENG exhibits an open–circuit voltage of 18 V and a short–circuit current of 1.05 μA, with a maximum power density of 28 W m^−3^ across a load resistor of 1 MΩ. The PENG has a long–term stability under a consecutive piezoelectric test for three hours. Later, Yadav et al. fabricated a flexible self–poled PENG using hexagonal boron nitride (hBN) nanoflakes embedded in PVDF with an open–circuit voltage, a short–circuit current, and a peak power density of 68 V, 0.1 µA, and 53.2 µW cm^−2^, respectively [80]. In addition, this PENG could perform robustly even after 45 days and 1500 bending periods. As mentioned above, Bagchi et al. reported a reusable self–poled PENG based on a MoS_2_/PVDF film, and the piezoelectric film exhibits a stable piezocatalytic dye degradation efficiency and could be used repeatedly at least 10 times [71].

## 8. Perspective

The low energy conversion efficiency of pure polymer materials has limited their application in energy harvesting. For this concern, it is a very promising design strategy for piezoelectric materials to synthesize composite materials with NFs. Under this circumstance, PVDF and its copolymers have high piezoelectric strain constants and are suitable materials for matrices. What is more important, the addition of NFs can improve piezoelectric properties without affecting their flexibility. In this paper, the recent research progress in the enhancement of piezoelectric properties of PVDF–based PENGs is reviewed, with a focus on composites of functional materials. The underlying mechanisms behind property enhancements are explained in detail. With respect to the conversion ability from mechanical energy to electrical energy, the piezoelectric coefficient is improved by adding piezoelectric materials as nucleating agents, which makes the composite material generate more β phase. It is foreseeable that with the improvement of properties of flexible PVDF piezoelectric materials and the progress of device–manufacturing technology, flexible PVDF piezoelectric materials will be widely used. Despite the considerable progress in the development and design of various PVDF functional devices, there are still many prominent problems to solve, and more work needs to be performed in the following aspects:

(1) The piezoelectric effect in PVDF is improved after other functional materials are added, generally because of the increase of the content of the β phase. It is necessary to gain a deeper understanding into the mechanism of how different NFs induce the compounds to form a β phase.
(2) Although at present there are many studies, the comparison of properties has not achieved the true uniformity of parameters, such as thickness, area, density, mass and operation method, and there is a lack of a unified standard which allows us to truly understand the property enhancement.
(3) In addition to incorporating NFs into the PVDF matrix, we should also pay attention to the innovative designs of the device structure. A novel structural design can not only promote the sensitivity of nanogenerators, but also satisfy other needs, such as wearable electronics in the field of textiles.

In summary, this review can help people engaged in the research field of PVDF–based PENGs to gain a comprehensive understanding into the latest progress and explore future research directions. It is foreseeable that these flexible and efficient PENGs will be applied to our daily life in the near future and offers a far–reaching impact on portable devices, wearable electronic devices, and biomedicine.

## Figures and Tables

**Table 1:** Performance of several poly(vinylidene fluoride) (PVDF)–based metal halide perovskite piezoelectric nanogenerators (PENGs).

Materials	Preparation Methods	Sample Size	Poling	Force or Pressure	V_oc_	I_SC_/J_SC_	Power@ Resistive Load (RL)	Refernece
FAPbBr_3_NPs/PVDF	Solution casting	1.2 × 1.4 cm^2^	50 kV cm^−1^	0.5 MPa	30 V	6.2 μA cm^−2^	-	[39]
MAPbI_3_/PVDF	Spin coating	1 × 1 cm^2^	80 kV cm^−1^	50 N	9.43 V	0.76 µA cm^−2^	-	[40,41]
MAPbI_3_/PVDF	Solution casting	-	Not supplied	2 kPa	1.8 V	37.5 nA	2.5 µW cm^−2^@0.6 MΩ	[41]
MAPbBr_3_/PVDF	Electrospinning	2.4 × 1.5 cm^2^	Not supplied	9.8 kPa	5 V	60 nA	0.28 µW cm^−2^@0.7 MΩ	[42]
MASnI_3_/PVDF	Spin coating	1 × 1 cm^2^	60 kV cm^−1^	0.5 MPa	12.0 V	4.0 μA cm^−2^	22 µW cm^−2^@3 MΩ	[43]
CsPbBr_3_/PVDF	Solution casting	-	Not supplied	100 MPa	120 V	35 μA	30 µW@3 MΩ	[44]
CsPbBr_3_@PVDF fibers	Electrospinning method and in situ growth	0.80 cm^2^	5 kV	-	103 V	170 µA cm^−2^	-	[46]

**Table 2:** Performances of the PVDF-based metallic oxide perovskite PENGs.

Materials	Preparation Methods	Sample Size	Poling	Force or Pressure	V_oc_	I_SC_/J_SC_	Power@RL	Ref.
PZT/PVDF	Solution casting	2 × 0.9 cm^2^	Not needed	8.5 kPa	55 V	-	35 µW cm^−2^@10 MΩ	[47]
PZT/PVDF	Hot pressing	-	Not needed	85.6 kPa	2.51 V	78.43 nA	-	[48]
BaTiO_3_ NPs/PVDF	Solution casting	1 × 1 cm^2^	2 kV	10 MPa	150 V	1500 nA	-	[49]
BaTiO_3_ NPs/P(VDF-HFP)	Spin coating	4 cm^2^	100 kV cm^−1^	0.23 MPa	75 V	15 μA	-	[53]
BaTiO_3_ NPs/P(VDF-TrFE)	Spin coating	-	Not needed	50 N	13.2 V	0.33 μA cm^−2^	12.5 µW cm^−2^@4 MΩ	[34]
BaTiO_3_ NPs/PVDF	Solution casting	1.5 × 2 cm^2^	Not needed	1 N	6.7 V	2.4 μA	-	[50]
BaTiO_3_ NPs/P(VDF-TrFE)	3D printing	-	50 V µm^−1^	60 N	6 V	2 μA cm^−2^	2 µW cm^−2^@10 MΩ	[51]
PMMA@BaTiO_3_ NW/P(VDF-TrFE)	Electrospinning	2.5 × 2.5 cm^2^	Not supplied	-	12.6 V	1.30 μA	4.25 μW@7.2 MΩ	[17]
BaTi_2_O_5_/PVDF	Hot pressing	4 × 2 cm^2^	Not needed	-	26 V	-	4.1 μW@22 MΩ	[33]
graphene/BaTiO_3_/PVDF	Electrospinning	2.5 × 2.5 cm^2^	12 kV	-	11 V	-	4 μW @ 8 MΩ	[16]
polydopamine/BaTiO_3_/P(VDF-TrFE)	Electrospinning	2.5 × 2.5 cm^2^	25 kV	700 N	6 V	1.5 μA	0.85 µW cm^−2^@4 MΩ	[54]
Ag/polydopamine/BaTiO_3_/PVDF	Selective laser sintering	0.9 × 1 cm^2^	Not supplied	-	10 V	142 nA	-	[55]
BaTi_0_._9_Zr_0_._1_O_3_/PVDF	Solution casting	2.5 × 2.5 cm^2^	8 kV	11 N	11.9 V	1.35 μA	0.15 µW cm^−2^@10 MΩ	[52]
KNN nanorods/PVDF	Electrospinning	2 × 2 cm^2^	20 kV	1 kPa	17.5 V	0.522 μA	-	[58]
silane/KNN nanorods/PVDF	Electrospinning	22 cm^2^	20 kV	1.1 kPa	21 V	22 μA	-	[59]
(1 − *x*)K_0.5_Na_0.5_NbO_3–x_BaTiO_3_/PVDF	Solution casting	3 × 3 cm^2^	10 kV	0.4 N	100 V	225 nA	1.4 µW cm^−2^@100 MΩ	[60]

**Table 3:** Performances of the PVDF-based PENGs doped with semiconductor piezoelectric materials.

Materials	Preparation Methods	Sample Size	Poling	Force or Pressure	V_OC_	I_SC_/J_SC_	Power@RL	Reference
GO/P(VDF-TrFE)	Solution casting	-	30 MV m^−1^	0.32 MPa	4 V	1.88 μA	4.4 µW cm^−2^@1 MΩ	[61]
rGO/PVDF	Electrospinning	-	Not supplied	20 kPa	-	0.5 μA	-	[62]
ZnO/PVDF	Solution casting	8 × 8 mm^2^	Not needed	28 N	24.5 V	1.7 μA	32 mW cm^−3^	[65]
BNNTs/P(VDF-TrFE)	Solution casting	1 × 1 cm^2^	10 MV m^−1^	0.4 MPa	22 V	600 nA	11.3 μW cm^−2^@6 MΩ	[31]
GeSe NSs/P(VDF-TrFE)	Solution casting	1 × 1 cm^2^	70 MV m^−1^	50 N	17.58 V	1.14 μA	9.5 μW cm^−2^@4 MΩ	[70]
MoS_2_/PVDF	Solution casting	1 × 1 cm^2^	Not needed	27.5 N	84 V	3.05 μA	47 mW cm^−3^@30 MΩ	[71]
